# Adapting a Complex Health Intervention Guided by the Core Functions and Form Model: A Methodology and Case Example of Participatory Adaptation

**DOI:** 10.21203/rs.3.rs-9587581/v1

**Published:** 2026-06-05

**Authors:** Kirsten Austad, Nhi Nguyen, Elizabeth Jadovich, Bridget Poznanski, Max Laguerre, Alicia Fernandez, Mari-Lynn Drainoni, Brian Jack, Suzanne Mitchell, Ramzi G. Salloum, Graham Moore

**Affiliations:** Boston Medical Center & Boston University Chobanian & Avedisian School of Medicine; Cambridge Health Alliance; Boston College; Boston Medical Center & Boston University Chobanian & Avedisian School of Medicine; Boston Medical Center & Boston University Chobanian & Avedisian School of Medicine; University of California, San Francisco; Boston University Chobanian & Avedisian School of Medicine; Boston Medical Center & Boston University Chobanian & Avedisian School of Medicine; UMass Chan Medical School; University of Florida College of Medicine; Cardiff University

**Keywords:** Intervention adaptation, Complex intervention, Hospital discharge, Non-English language preference, Core Functions and Forms

## Abstract

**Background:**

Adapting evidence-based interventions (EBIs) to fit local contexts while preserving fidelity to core mechanisms is challenging, particularly for complex, multicomponent interventions. The Core Functions and Forms (CFF) model distinguishes essential intervention mechanisms (core functions) from adaptable delivery strategies (forms), offering a principled approach to balance fidelity and tailoring. We applied CFF to adapt hospital discharge EBIs for patients with non-English language preference (NELP).

**Methods:**

Using Steps 1 and 2 of the ADAPT framework integrated with CFF, we convened an Adaptation Team to (1) rate the importance of core functions for NELP discharge care, (2) generate and evaluate forms—divided into specific forms (concrete components) and cross-cutting forms (e.g., who delivers, when, where, and how)—and (3) refine a pilot intervention. We collected participant demographics, audio-recorded and transcribed four facilitated meetings, and administered structured ranking and rating surveys via REDCap. Two analysts conducted rapid content extraction within one week of each meeting to compile ideas for subsequent rounds. Quantitative synthesis included mean importance rankings and mean impact and feasibility scores (1–10 scale). Specific forms were catalogued by corresponding core function.

**Results:**

The Adaptation Team (n=13) included clinicians, nurses, professional interpreters, social workers, and patient/caregiver representatives with lived NELP experience. The highest-ranked core functions were “understand and address patient priorities” and “assess and address linguistic and cultural needs.” For intervention delivery, physicians were rated highest for impact (mean=9.5) but lowest for feasibility (mean=4.8), while nurses, social workers, and community health workers offered better balance with high impact (mean range 8.1–8.5) and greater feasibility (mean range 7.3–7.4). In-person contact during admission plus post-discharge follow-up was rated most impactful (mean=9.4) but least feasible (mean=4.3), though feasibility improved with remote delivery (mean=8.0). For language concordance, remote delivery by a language-concordant provider best balanced impact (mean=8.1) and feasibility (mean=8.3).

**Conclusions:**

This work demonstrates operational integration of CFF into ADAPT, coupling theory-driven function specification with systematic stakeholder engagement. Key innovations include applying CFF across a family of related discharge EBIs to enable reuse of shared mechanisms and explicitly separating specific from cross-cutting forms to highlight implementation-relevant decisions.

## BACKGROUND

Intervention adaptation, the intentional modification of a program, practice, or innovation’s design or delivery, is a common strategy to improve an intervention’s fit within specific contexts (1). When evidence-based interventions (EBIs) are implemented with diverse populations, adaptation plays a crucial role in aligning interventions with the unique needs and preferences of these groups (2–4). This alignment can enhance an intervention’s implementability and effectiveness and promote health equity, making adaptation a vital component of efforts to achieve equitable health outcomes. (5–8) Despite its importance, many questions remain about the best approaches to adapting interventions, particularly in high population diversity contexts (9,10).

Ensuring an EBI fits the local context is a widely accepted first step in implementation. Frameworks such as ADAPT and ADAPT-ITT explicitly require assessing intervention-context fit and recommend that adaptations remain aligned with the intervention’s functions (11–13). A persistent tension is tailoring interventions while ensuring that effect-producing elements are preserved. Frameworks such as Consolidated Framework for Implementation Research (CFIR) distinguish “core components” versus “adaptable periphery” (14–18). However, in practice it is difficult to specify which elements are truly essential or to predict which adaptations will alter an intervention’s mechanism of action or have unintended effects (19–21). This uncertainty is greater for complex interventions, those that feature multiple interacting components, variable implementation processes, heterogeneous outcomes, and complex interactions with context, because it is harder to measure and track which elements are responsible for the observed effects (22–24).

One approach that may address these challenges is the Core Functions and Forms (CFF) model (25–27). The CFF operationalizes ideas first advanced by Hawe and colleagues in 2004 around defining intervention integrity in terms of functions, while allowing forms to vary across context.(25) CFF posits that interventions are defined by a consistent set of core functions (the essential mechanisms of an intervention to preserve), while the forms (the specific ways these functions are delivered) can and should vary based on the needs of the implementation context and population. For example, consider an evidence-based patient education intervention originally developed and tested using an English-language handout. The core function of the EBI is “to help patients understand their illness.” If one sought to adapt the EBI for a setting where many patients prefer Spanish or have limited literacy, the same function would not be achieved by an English language handout but would require a written Spanish handout, illustrated materials, or a language-concordant audio recording of the content of the written materials. Not adapting the form—e.g., providing only English text because of concerns that changes might weaken the intervention—would in fact undermine its effectiveness. By centering adaptation on functions rather than fixed components, CFF offers a principled way to resolve the fidelity-tailoring paradox which aims to preserve the mechanisms that produces benefits (the function), while permitting flexible, context-appropriate changes in delivery (the form).

A deeper understanding of these core functions and adaptable forms offers a useful conceptual model for equity-focused intervention adaptation. This manuscript aims to describe the application of the CFF model to support stakeholder-engaged intervention adaptation and analyze the benefits and drawbacks of this approach. We focus on a case study involving the adaptation of a hospital discharge EBI for patients with a U.S. safety net hospital. Specifically, we applied the CFF model within the ADAPT process to define core functions and identify context-appropriate forms for hospital discharge interventions serving NELP populations (28).

### Case Example: Evidence-Based Intervention to Improve Hospital Discharge

The transition from hospital to home after hospital discharge is a high-risk and vulnerable period for both patients and caregivers (29). Hospital discharge processes require coordination among hospital teams, clear handoffs between inpatient and outpatient clinicians, accurate medication reconciliation, and instructions to patients and caregivers that are both understandable and actionable. Undelivered or unclear communication, ambiguous delineation of responsibilities, and unrealistic expectations placed on patients and their caregivers (30–32) are common and contribute to high readmission rates and low patient satisfaction (33). Several hospital discharge interventions have been shown to reduce hospital readmission rates and improve patient-centered outcomes, including Project RED, Care Transitions Intervention, and Project BOOST (34–39). These hospital discharge EBIs all exemplify complex interventions because they include multiple interacting components, generate non-linear causal pathways, vary substantially in how they are implemented across settings, and are contextually dependent.(40)

An important limitation, however, is that nearly all hospital discharge EBIs have been designed for and tested only in English-fluent populations (41,42). Individuals with non-English language preference (NELP), a growing demographic in the United States over the past 40 years (43), have been routinely excluded from hospital discharge trials yet are at very high risk for poor outcomes due to inherent communication barriers.(44,45) As a result, these EBIs do not address language-related barriers that arise in routine practice, such as translating provider-written discharge instructions or ensuring a qualified interpreter is present for discharge teaching.(46,47) Without deliberate adaptation, deploying these EBIs in real-world, linguistically diverse settings risks reducing their effectiveness and widening health inequities (48–50). Because discharge EBIs are complex and overlapping, a function-based approach is particularly useful for ensuring essential mechanisms remain intact when adapting for linguistically diverse populations.

## METHODS

### Overview of Approach

**Figure 1:**
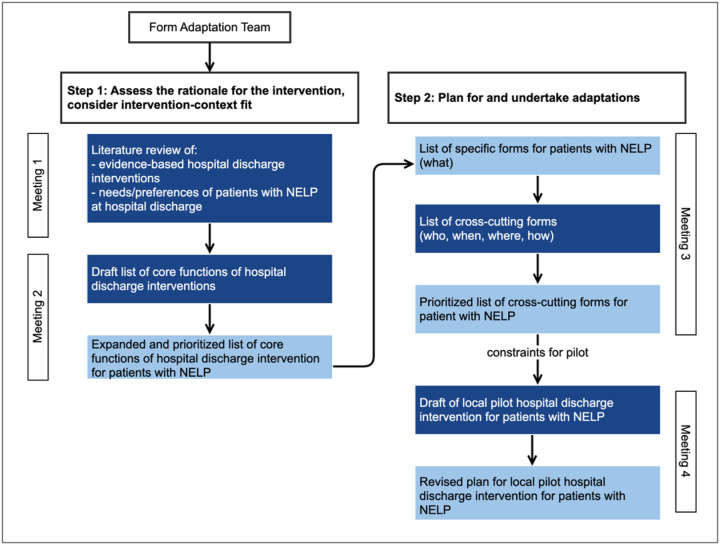
In depth step-by-step process of ADAPT step of mapping similarities and differences between the new context and adapted context, which was operationalized using the Core Functions and Form Model. Boxes in purple represent tasks led by the Principal Investigator while boxes in blue indicate those led by the Adaptation Team

The overall goal of this study was to adapt a hospital discharge support for patients with non-English language preference (NELP) and to pilot test it at a single safety net academic medical center. The author team selected the ADAPT framework to guide the process, with some tasks divided between the author group and the Adaptation Team (denoted by colors in Figure 1) (11). Our assessment of intervention-context fit (discussed above) found that no single existing hospital discharge intervention would fit the needs of populations with NELP. Rather than adapting a single hospital discharge EBI, we noted that many hospital discharge EBIs share the same components, such as patient education, medication reconciliation, care coordination, and post-discharge outreach.(39,40) Accordingly, we identified and ranked the core functions shared across these EBIs, prioritizing those most pertinent to NELP patients. Adaptation then focused on designing or selecting context-appropriate forms—specific activities, materials, and delivery strategies—that could reliably achieve those core functions in a multilingual, multicultural care setting. Framing adaptation around functions (what must be achieved) rather than forms (which specific program components to change) aimed to preserve the mechanisms that produce benefit while tailoring delivery to the needs of NELP patients.

**Figure 2:**
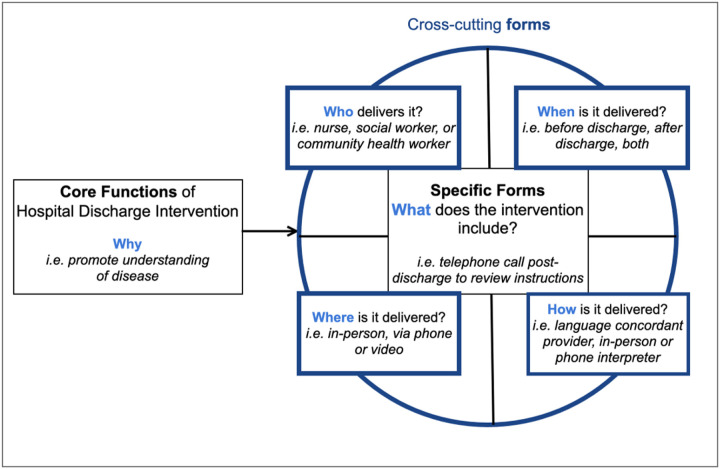
Core Functions and Forms Model applied to adaptation of a hospital discharge intervention for patients with non-English language preference (NELP)

Once formed (Figure 1), the Adaptation Team was given three tasks: (1) identify and prioritize the core functions of a hospital discharge intervention for NELP patients; (2) propose context-specific adaptable forms for delivering each core function, distinguishing between *specific forms* (what is delivered) and *crosscutting forms* (who delivers it, where and when it is delivered, and how language concordance is achieved in delivery; see Figure 2); and (3) provide iterative feedback on proposed functions and forms to inform pilot implementation.

### Study Setting

Study activities occurred at Boston Medical Center (BMC), the largest safety net hospital network in New England.(51) BMC serves a diverse population, with the majority of patients identifying as Black or Latinx. Approximately one-third of all patients have NELP. This high linguistic diversity context includes a patient population that is about 54% Spanish speaking, 18% Haitian Creole speaking, 9% Cape Verdean (Portuguese) Creole speaking, 7% Vietnamese speaking, and the remainder speaking over 250 additional languages.

### Participant Recruitment

We aimed to recruit 10–14 Adaptation Team members, including patient and caregiver representatives and BMC employees. Patient and caregiver representatives were eligible if they had direct experience with hospital discharge as a patient with NELP or had assisted a patient with NELP during discharge. We prioritized recruiting individuals whose first language was not English. To identify these representatives, we engaged community networks and used snowball sampling to reach individuals outside BMC with relevant lived experience. We provided simultaneous interpretation and translation services to enable participation by monolingual individuals with NELP. Non-employee participants received $125 pre-loaded gift cards for each adaptation meeting attended and for completing voting and survey activities.

We sought to include BMC employees across provider types involved in hospital discharge, including physicians, advanced practice providers, nurses, case managers, and social workers. To recruit BMC staff, we emailed supervisors in nursing, interpreter services, social work, and inpatient provider departments, who then shared study information with potentially interested team members.

### Adaptation Team Meetings

We convened the Adaptation Team via videoconference four times (May–June 2025). The PI facilitated using slides plus interactive Mentimeter polls and comment boxes to encourage participation.(52) Table 2 summarizes meeting topics. Meeting 1 opened with introductions and a process -mapping exercise to describe the hospital discharge process from an NELP patient’s perspective, followed by a brief literature summary of discharge EBIs and a group brainstorming of challenges, strengths, and implications for intervention design. Each session began with a short recap and open discussion. In Meeting 2 we presented a revised process map, introduced the CFF model, and reviewed an initial literature-derived list of candidate core functions (limited to EBIs feasible without repeated home visits). Team members drew on clinical and lived experience to prioritize, critique, and add functions. In Meeting 3 the- group rated impact and feasibility of functions via Mentimeter polls and then generated intervention forms. Forms were categorized into two types (Figure 1): *specific forms* that correspond to individual core functions, and *cross-cutting forms* that operationalize multiple core functions, such as who delivers the intervention, where, when, and how (particularly in terms of addressing language barriers). For specific forms, participants brainstormed as many ideas as time allowed. For the cross-cutting forms, the research team provided a list of options and asked participants to begin considering the feasibility and impact of each using Mentimeter polls, which laid out the groundwork for the formal voting and ranking process described below. Meeting 4 presented voting results, facilitated discussion, and solicited feedback on the proposed pilot model based on informed collected in earlier meetings within the constraints of alignment with the funded grant.

### Data collection

Demographic information for each Adaptation Team member was collected using a REDCap survey. All four meetings were audio-recorded and transcribed using Zoom’s AI transcription feature. Transcripts were then reviewed by a research assistant (RA) for accuracy and identifying information was redacted to maintain participant confidentiality.

Rating by all Adaptation Team members was conducted using REDCap surveys. Members ranked a list of 15 core functions of a hospital discharge intervention for patients with NELP on a scale from 1 to 15, where 1 indicated the core function with the highest importance and 15 the lowest. Additionally, for each core function, members assigned two scores from 1 to 10: one representing the perceived impact of the core function and the other representing the feasibility of incorporating it into a hospital discharge intervention. Lower scores indicated lower impact or feasibility, respectively.

The Adaptation Team also rated the impact and feasibility of cross-cutting forms, Participants were asked to assign scores from 1 to 10 to indicate the perceived feasibility and impact across the following questions: where and when the intervention should be delivered, who should deliver the intervention, and how to overcome the language barrier.

### Data Analysis

Two analysts independently reviewed the transcripts from each meeting. We used conventional content analysis with descriptive coding to extract and enumerate discrete ideas or suggestions from participants. Prior to the next meeting, these ideas were compiled into a consolidated list, which served as the basis for subsequent discussion and rating.

The research team then conducted summative analyses of the Adaptation Team’s input on core functions. From the core function ranking exercise we calculated an average ranking to establish a consensus order of overall importance. Next, we computed mean impact and mean feasibility scores from the voting exercise. We produced a bubble plot to display these results with mean feasibility on the x-axis, mean impact on the y-axis, and one bubble per core function; bubble size represented standard deviation, so larger bubbles indicated greater variance in opinions. This visualization highlighted functions that combined high impact with high feasibility, as well as those judged high impact but less feasible to implement. We applied the same analytic approach to each of the crosscutting forms, generating analogous bubble plots to summarize perceived impact–feasibility tradeoffs for those elements. Lastly, we abstracted from transcripts all ideas for specific forms generated by the Adaptation Team and organized them by corresponding core function.

### Ethics

This study was approved by the Boston University Medical Center Institutional Review Board (H-44249).

## RESULTS

### Adaptation Team and Meeting Structure

In total, 13 individuals participated in the Adaptation Team. Participant demographics are provided in Table 1. All participants were fluent in English, 69% spoke at least one additional language and 54% spoke a language other than English at home. Most team members (77%), including non-BMC employees, were employed in health-care related fields. The team was made up of primarily females (92%) who were young adults 35 years or under (69%) and had previously or currently acted as caregiver for a loved one (85%).

Initially we planned to conduct voting during Meetings 2 and 3 using Mentimeter. However, based on participant feedback we moved to asynchronous —distributing polls via email after meetings and allowing members several days to respond— after Meeting 3 (Table 2). Attendance was relatively consistent throughout the course of the adaptation process with the number of Adaptation Team members in attendance ranging from 8 to 11.

### Ranking of Core Functions

In the ranking exercise, participants ranked “understanding and addressing priorities of the patients” as the most important core function (mean rank =13.7, out of 15) followed by “assess and address linguistic and cultural needs” (mean rank =11.8) and “involve caregiver” (mean rank =10.9). Lower ranked core functions included “promoting self-care” (mean rank = 5.1), “ensure transmission of information to primary care provider” (mean rank =4.3), and “promote inpatient continuity” (mean rank =2.1).

### Rating Core Functions

**Figure 3:**
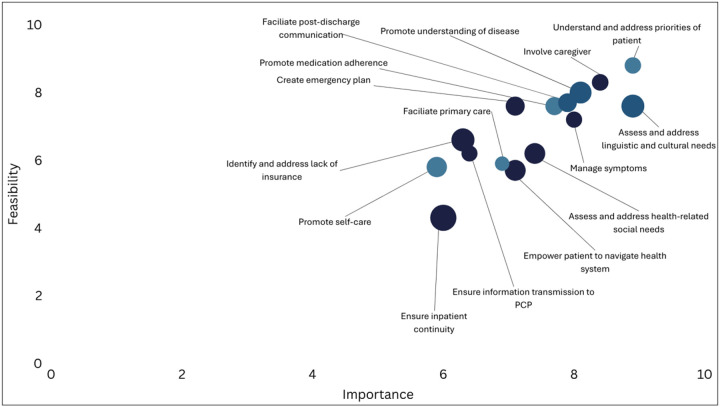
Bubble quadrant plot of feasibility and importance rankings for core functions of hospital discharge interventions for patients with non-English language preference (NELP). Mean feasibility is on the x-axis, mean impact on the y-axis, and one bubble per core function. Bubble size represented standard deviation, so larger bubbles indicated greater variance in opinions

When Adaptation Team members rated the impact of each core function, “understand and address patient priorities” and “involve caregivers” received the highest impact ratings (mean impact score = 8.9 for both, out of 10) and were also rated as highly feasible (mean feasibility scores = 8.8 and 8.3, respectively). The next most impactful core function was “assess and address linguistic and cultural needs” (mean impact score = 8.4), but slightly less feasible (mean feasibility score = 7.6). Core functions rated as having low feasibility of achieving included ensuring inpatient continuity” (mean feasibility score = 4.3), “empower patient to navigate the health system” (mean feasibility score = 5.7), and “promote self-care” (mean feasibility score = 5.8). These results are summarized in the bubble plot in Figure 3 (ranking in Appendix).

### Rating Forms

**Figure 4:**
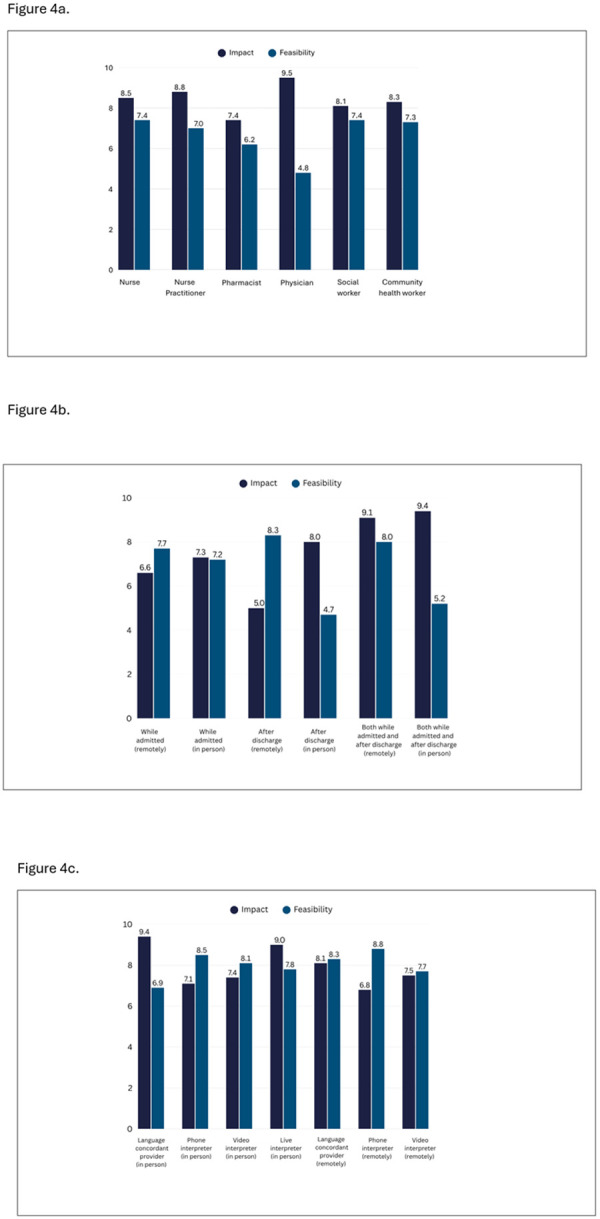
Feasibility and impact ratings of cross-cutting forms of hospital discharge interventions for patients with non-English language preference (NELP)

For cross-cutting options on who should deliver the intervention (Figure 4a), physician delivery was rated highest for perceived impact (mean impact = 9.5) but lowest for feasibility (mean feasibility = 4.8). Nurses, social workers, and community health workers were also seen as highly impactful (mean impact range 8.1–8.5) while being considerably more feasible (mean feasibility range 7.3–7.4), suggesting these non-physician roles offer a better balance of effect and practicality.

For decisions about timing and location of delivery (Figure 4b), Adaptation Team members rated in--person approaches as having greater impact than remote modalities, and models that included contact both during the hospital stay and after discharge as more impactful than those limited to only one time period. The highest impact model was in-person contact during admission plus post--discharge follow--up (mean impact = 9.4), but it scored low on feasibility (mean feasibility = 4.3). By contrast, a remote-only post--discharge- model (in which interventionists connect with patients via phone or video rather than in person) received the highest feasibility rating (mean feasibility = 8.3) but a lower impact score (mean impact = 6.6), illustrating a clear tradeoff between expected impact and practicality.

For crosscutting options for achieving language concordance with diverse NELP participants (Figure 4c), team ratings favored in-person, language-concordant delivery as most impactful (mean impact = 9.4), closely followed by in-person delivery using a live interpreter (mean impact = 9.0). Phone delivery by a language concordant provider was rated as less impactful than in-person care (mean impact = 8.1) but more feasible (mean feasibility = 8.3). Remote delivery using an interpreter by phone or video scored lower on impact (mean impact = 6.8 and 7.5, respectively), indicating that language-concordant providers—especially in person—were seen as the most effective option, while telephone delivery by a concordant provider offered the best balance of impact and feasibility.

### Intervention Forms

Table 3 links the specific forms suggested by the Adaptation Team for four of the Core Functions. For understanding and addressing patient priorities, examples include structured tools (such as a goalsetting worksheet or a strengths/needs assessment) and standardized documentation of patients’ top three priorities and linguistic/cultural preferences. To promote disease understanding, suggested forms include language-concordant written instructions, links to educational videos in preferred language, peer educators, and post-discharge phone calls to reinforce instructions. To involve caregivers, recommendations focused on documenting the primary caregiver and scheduling discharge teaching when the caregiver can attend. To facilitate post-discharge communication, proposals included providing a direct post-discharge phone line, creating an online question forum, enrolling patients in the patient portal during admission, and offering hospital lobby computers for messaging access.

## DISCUSSION

The need for improved approaches to adapting complex multi-component interventions is well documented (53). In this manuscript we describe how the CFF model, integrated with the ADAPT methodology, guided our adaptation of hospital discharge interventions to better meet the needs of patients with NELP (26,28). Framing adaptation around core functions (the causal processes that must be preserved to achieve intended effects) and adaptable forms (the context-sensitive ways to deliver those core functions) allowed us to translate shared functions across hospital discharge EBIs into delivery options tailored to those with NELP patients.

Although interest in CFF is growing, prospective, stakeholder engaged applications remain -limited.(54–57) Unlike prior applications that described adaptations retrospectively, we developed a stepwise, prospective process for engaging stakeholders to identify and prioritize core functions and select context appropriate- forms (58–62). In this way, CFF complements ADAPT: ADAPT provides the systematic steps for assessing fit and documenting changes, while CFF supplies explicit guidance on what to preserve (functions) versus what can be varied (forms such as activities, materials, and delivery modes) to maintain mechanisms of effect across contexts.(11,63)

A novel feature of our methodology is applying CFF across a family of related EBIs rather than to a single program. We treated intervention adaptation and development as a continuum: infer core functions from existing hospital discharge interventions, prioritize those most likely to benefit the new target population, and then choose context-appropriate forms to deliver those functions. This function-to-form approach shows how shared mechanisms across different EBIs can be reused and tailored, enabling coordinated, scalable adaptations for interventions with common goals and components. After piloting the adapted intervention, we plan a hybrid effectiveness–implementation trial to confirm clinical benefit and continue evaluating implementation outcomes. Because our adaptation combines mechanisms and delivery features from multiple programs, such a trial is warranted in this case, but may not be in another application of the methodology where adaptations are less transformative.

To enhance operational usefulness, we subdivided forms into specific forms and crosscutting forms. This clarifies “form” by separating what is delivered from who delivers it, when/where, and how. Specific forms are concrete components (e.g., written discharge summaries, post- -discharge calls); crosscutting forms are implementation features that span components (e.g., deliverer, timing, communication mode, language modality). This distinction was useful where a single core function could be achieved via multiple component-–delivery combinations. Making specific and crosscutting forms explicit highlighted implementation decisions often omitted from intervention descriptions and prompted systematic consideration of linguistically relevant adaptations (e.g., in- person vs remote delivery and how to achieve language concordance). These choices often determine -whether an intervention is accessible and effective for NELP patients.

A key drawback of CFF is that it does not systematically prompt adapters to anticipate unintended consequences of changing forms. Frameworks such as Model for Implementation Design and Impact (MADI) emphasize mapping downstream “ripple” effects and documenting potential mediators and moderators when delivery features are altered.(21) For example, replacing inp erson, language-concordant delivery with interpreter- mediated- phone encounters may still convey key discharge information (core function), but can also reduce patient engagement, erode trust, and limit nonverbal communication.(64) Those downstream effects may blunt the intervention’s mechanism (e.g., patient activation or comprehension) and ultimately reduce its effectiveness counter to the intention of adaptation efforts. When correctly applied the CFF should help adapters know through measurement of core functions if it was achieved or not; without complementary attention to causal pathways it may not explain why a form change produced that failure.

A central challenge for CFF and other mechanism focused adaptation frameworks is reliably identifying the mechanisms themselves. Guidance calls for explicit attention to theory or mechanisms of action and for preserving “core components,” yet many trial-s do not define or justify those elements (1). Mechanisms of hospital discharge interventions are poorly specified. In our case study we relied on a preliminary list of core functions was derived from a structured literature review. An alternative and more rigorous approach would be to use a formal consensus process (e.g., Delphi) to engage a broad group of experts in hospital discharge intervention including social workers, physicians, and nurses to define functions (60,65). As the field advances, aligning terms such as “core components,” “mechanisms,” and “core functions” across intervention development, implementation science, and adaptation will improve consistency and guide methodological choices (55,66,67).

An additional limitation concerns the level of abstraction at which core functions should be specified, as prior applications of the CFF model have varied in granularity. For example, in applying CFF to the patient-centered medical home, Perez Jolles et al nested multiple core functions within an overarching principle (e.g., “accessible care”)(68). In our application, some elements, such as “involve caregivers,” could reasonably be conceptualized as higher-level principles rather than core functions, with more specific operationalizations articulated beneath them. Alternative structuring choices may have resulted in a different set or hierarchy of core functions. Future work should further clarify guidance on how to define and operationalize core functions to enhance consistency and comparability across studies.

The approach we present here has important implications for health equity. Patients with NELP face many barriers to EBIs and have disproportionately high risk of communication failures at discharge and adverse outcomes such as unplanned readmissions (33,49), yet have largely been excluded from prior discharge trials (41,42). Creating entirely new interventions for each language group would be costly and underuse existing evidence.(69) Health disparities scholars have warned of “sideways progress,” where repeated, intensive adaptations for each demographic subgroup consume resources without producing broadly scalable solutions (70). Our approach seeks efficiency and real-world- relevance by (1) building on proven discharge EBIs and making targeted adaptations needed for NELP populations and (2) adapting for the broader NELP group rather than designing separate programs for each language or cultural subgroup, aligning adaptations with diverse clinical settings to improve feasibility and scalability (10).

## CONCLUSION

Integrating the CFF model with the ADAPT process offers a practical and theory-grounded approach for equity-focused adaptation of complex, multicomponent interventions and effectively facilitated adaptation by drawing from the numerous hospital discharge interventions tested to date. By specifying measurable core functions and distinguishing between specific and cross-cutting forms, this combined approach helps implementers preserve intervention mechanisms while tailoring delivery to the linguistic and cultural needs of populations with NELP. Applying these methods to a family of related discharge interventions, rather than to a single program, illustrates how shared mechanisms can be translated into context-sensitive delivery options that are both actionable for health systems and amenable to rigorous evaluation. Future work should refine guidance on defining core functions and test this approach across diverse settings and populations.

## Supplementary Material

Supplementary Files

This is a list of supplementary files associated with this preprint. Click to download.

- Appendix.docx

## Figures and Tables

**Table 1:** Demographics of Adaptation Team Members

Demographic	N (%)
Gender	
Male	1 (8)
Female	12 (92)
Age	
18–24 years	3 (23)
25–35 years	6 (46)
36–45 years	3 (23)
46–55 years	0 (0)
55 years or older	1 (8)
Profession	
Health Care Worker: physician, advanced practice provider, or resident	4 (31)
Health Care Worker: Interpreter	2 (15)
Health Care Worker: Social Worker	3 (23)
Health Care Worker: Other	1 (8)
Other	3 (23)
Languages of Fluency	
English	13 (100)
Spanish	5 (38)
Haitian Creole	3 (23)
Vietnamese	1 (8)
French	1 (8)
Language Spoken Most Often at Home	
English	6 (46)
Spanish	3 (23)
Haitian Creole	2 (15)
Vietnamese	2 (15)
Ever Been Hospitalized	
Yes	8 (62)
No	5 (38)
Ever Acted as a Caregiver	
Yes	11 (85)
No	2 (15)
Boston Medical Center Employee	
Yes	7 (54)
No	6 (46)

**Table 2:** Overview of Adaptation Team Meetings and Activities

Date	Description	N	Goals and Agenda
05/06/2025	Meeting 1 Virtual	10	Introduction of meeting participants Build a shared understanding current discharge process (part I) Literature review on evidence-based hospital discharge interventions and patients with NELP at hospital discharge Brainstorm needs and strengths of patients with NELP relevant to hospital discharge
05/13/2025	Meeting 2 Virtual	11	Review and summarize of last session and interim input Build group understanding current discharge process (part II) Explanation of CFF model Brainstorm core functions of hospital discharge intervention
05/20/2025	Meeting 3 Virtual	8	Review and summarize of last session and interim inputBrainstorm forms of a hospital discharge intervention for patients with NELP including: Forms that correspond to a single core function (the “what”) Cross-cutting forms that specify the means of delivery of other forms (the “who, when, where, and how”)
	*Voting		Rank importance of core functions
	Asynchronous		Rate feasibility and impact of each core function Rate feasibility and impact of the crosscutting forms
06/02/2025	Meeting 4 Virtual	10	Review results of group voting Open discussion of proposed design of pilot intervention at Boston Medical Center

CFF = Core Functions and Forms model; NELP = non-English language preference

* Initially planned on voting during Meeting 2 and 3 however based on feedback from Adaptation Team members this was made asynchronous after Meeting 3 to allow further time for deliberation

**Table 3:** Example of specific forms suggested by Adaptation Team linked to core functions

Core Function	Specific Forms
Understand and address patient priorities	Create a document for patients to brainstorm health goals Include in standardized documentation patient's top three priorities Include in standardized documentation linguistic and cultural preferences Develop a “strengths and needs” patient assessment tool
Promote understanding of disease	Offer language concordant written discharge instructions Provide links to educational videos in preferred language Offer education from a peer Telephone call post-discharge to reinforce written discharge instructions
Involve caregiver	Document patient preferences around most important caregiver Schedule discharge teaching at a time caregiver can be present
Facilitate post-discharge communication	Provide a number to call after discharge that gives direct access to a provider Create an online post-discharge question forum Actively enroll patients in MyChart while admitted Have computers in hospital lobby for patients to use for post-discharge messaging in case they do not have access to technology

## Abbreviations

BMC: Boston Medical Center
CFF: Core Functions and Form
EBI: evidence-based intervention
MADI: Model for Implementation Design and Impact
NELP: non-English language preference
